# Supplementary material for: Splice-Junction-Based Mapping of Alternative Isoforms in the Human Proteome
Source: Cell Rep. Author manuscript; Available in PMC 2020 Jan 15. (PMC6961840; doi:10.1016/j.celrep.2019.11.026)
Supplement: 2 [file NIHMS1546469-supplement-2.zip › DF1/PXD010154/Colon/Colon_3_MAP4_AGQNDPLKDSYVPLELAK.pdf]

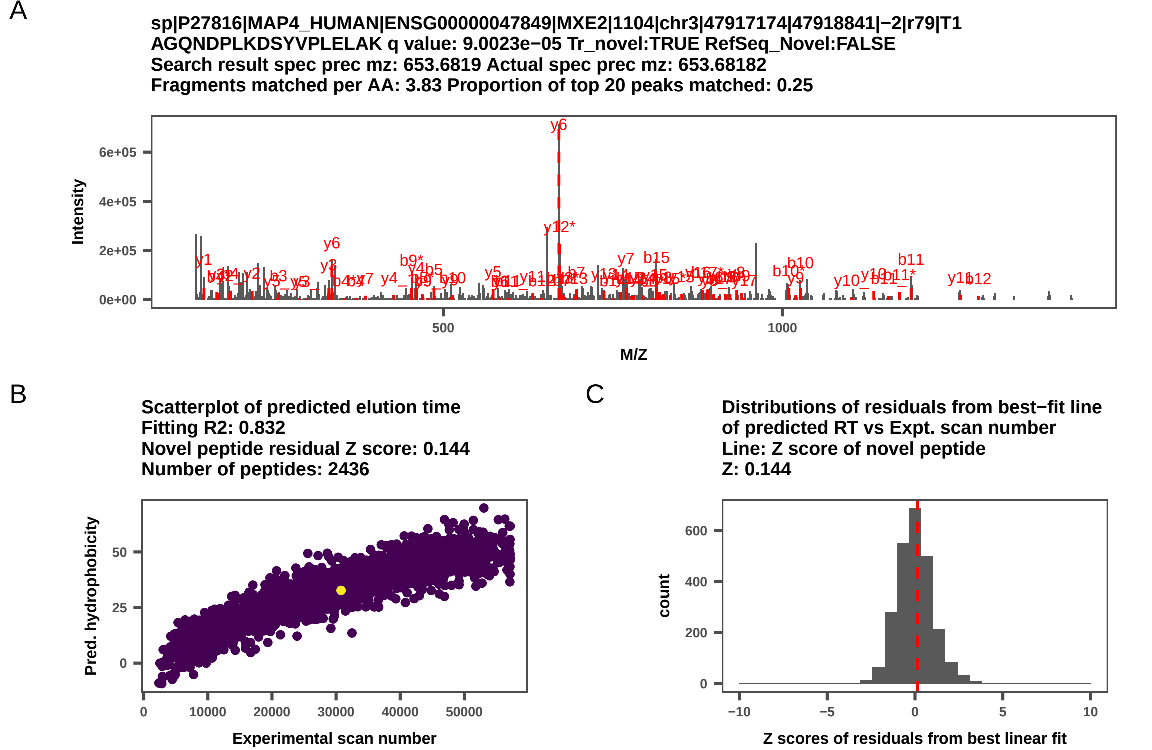
A
sp|P27816|MAP4_HUMAN|ENSG00000047849|MXE2|1104|chr3|47917174|47918841|−2|r79|T1
AGQNDPLKDSYVPLELAK q value: 9.0023e−05 Tr_novel:TRUE RefSeq_Novel:FALSE
Search result spec prec mz: 653.6819 Actual spec prec mz: 653.68182
Fragments matched per AA: 3.83 Proportion of top 20 peaks matched: 0.25
Intensity
6e+05
4e+05
2e+05
0e+00
500
1000
M/Z
y6
y12*
y1
y7
b15
b10
b11
b12
B
Scatterplot of predicted elution time
Fitting R2: 0.832
Novel peptide residual Z score: 0.144
Number of peptides: 2436
Pred. hydrophobicity
50
25
0
0
10000
20000
30000
40000
50000
Experimental scan number
C
Distributions of residuals from best−fit line
of predicted RT vs Expt. scan number
Line: Z score of novel peptide
Z: 0.144
count
600
400
200
0
−10
−5
0
5
10
Z scores of residuals from best linear fit
